# Uncovering Glucose-6-Phosphate Isomerase (GPI) Deficiency in a Five-Year-Old With Hemolytic Anemia in Bahrain

**DOI:** 10.7759/cureus.86331

**Published:** 2025-06-19

**Authors:** Maryam Busehail, Fajer N Qasim, Fatema J Alasheeri, Ameera Aloraibi

**Affiliations:** 1 Pediatrics, Salmaniya Medical Complex, Manama, BHR; 2 General Practice, Salmaniya Medical Complex, Manama, BHR; 3 Pediatric Hematology/Oncology, Salmaniya Medical Complex, Manama, BHR

**Keywords:** gpi deficiency, #hemolytic anemia, pediatric genetics, pediatric hematology, pediatrics, whole-exome sequencing

## Abstract

Glucose-6-phosphate isomerase (GPI) deficiency is a rare autosomal recessive enzymopathy that presents with chronic nonspherocytic hemolytic anemia. We report the case of a five-year-old Bahraini girl with a history of neonatal jaundice and recurrent episodes of anemia. A comprehensive diagnostic workup, including peripheral smear, bone marrow examination, and whole-exome sequencing, revealed a homozygous likely pathogenic variant in the GPI gene, confirming the diagnosis of GPI deficiency. The patient was managed with supportive measures including blood transfusions, folic acid, vitamin D supplementation, and iron chelation therapy. This case underscores the importance of considering rare inherited enzymatic disorders in the differential diagnosis of pediatric hemolytic anemia and highlights the critical role of genetic testing in achieving diagnostic clarity and informing long-term care.

## Introduction

Glucose-6-phosphate isomerase (GPI) deficiency is a sporadic, autosomal recessive disorder characterized by hemolytic anemia due to impaired glycolysis and gluconeogenesis. GPI catalyzes the isomerization of glucose-6-phosphate to fructose-6-phosphate, a fundamental step in energy production in red blood cells (RBCs). Deficiency of this enzyme results in RBC membrane instability, leading to chronic hemolysis caused by increased RBC turnover. Despite its rarity, GPI deficiency is a significant cause of hemolytic anemia, with other manifestations including jaundice, splenomegaly, and, in some cases, skeletal and neurological abnormalities [1].

GPI deficiency is often overlooked due to its rarity and clinical overlap with other hemolytic anemias (e.g., G6PD deficiency, hereditary spherocytosis); its diagnosis is confirmed through enzyme activity assays and genetic testing, identifying homozygous or compound heterozygous mutations in the GPI gene located on chromosome 19q13.1 [1]. Management is supportive, typically involving blood transfusions in severe cases [2]. Given its rarity, the disease remains under-recognized, and early diagnosis is crucial for appropriate patient care.

The disease can present in early childhood, with affected individuals experiencing recurrent episodes of hemolysis, often exacerbated by infection or other stressors. We report a case of a five-year-old female diagnosed with GPI deficiency, presenting with recurrent anemia exacerbations triggered by infections.

## Case presentation

The patient, a firstborn of a consanguineous marriage, was born at full term via normal vaginal delivery. She developed early neonatal jaundice (unconjugated type) requiring phototherapy within hours of birth. Laboratory workup revealed severe anemia (Hb 8 g/dL), necessitating blood transfusion and a two-week hospital stay. Post-discharge, she was followed by hematology for persistent hemolytic anemia requiring monthly transfusions.

**Figure 1:**
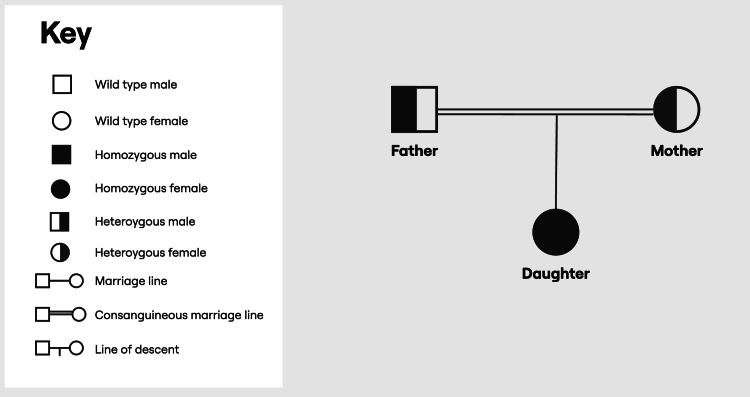
Family pedigree of one child with GPI Image credits: Maryam Busehail, Fajer Qasim, Fatema J. Alasheeri, Ameera A. Aloraibi GPI: Glucose-6-phosphate isomerase deficiency

Due to persistent anemia of unexplained etiology, whole-exome sequencing was performed at three years of age, revealing a diagnosis of GPI deficiency.

On physical examination, the child was pale, not icteric, with no lymphadenopathy. The systemic review was normal. Abdominal examination revealed a soft, lax abdomen, with hepatomegaly, around 2-3 cm below the right costal margin, and a palpable spleen around 3 cm below the left costal margin. The rest of the examinations were unremarkable.

A comprehensive series of laboratory investigations was conducted to support and confirm the diagnosis of GPI deficiency. These tests included a complete blood count (CBC), iron profile, liver function tests, peripheral blood smear, bone marrow aspiration, and genetic sequencing.

Detailed laboratory findings of the patient are shown in Table 1.

**Table 1:** Laboratory results of a patient with GPI deficiency GPI, glucose-6-phosphate isomerase; MCV, mean cell volume; MCH, mean cell hemoglobin; RBCs, red blood cells; MPV, mean platelet volume; WBCs, white blood cells

Laboratory test	Patient’s result	Normal range
Complete blood count		
Hemoglobin (g/dL)	8	12-14.5
Hematocrit (%)	29.20	33-45
MCV (fL)	98.3	80-97
MCH (pg)	30.1	27-33
RBCs (cells/)	2.97	3.9-5.2
Platelets (x10^9^/L)	300	150-400
MPV (fL)	11.3	8-11.5
WBCs (x10^9^/L)	11.54	3.6-9.6
Neutrophils (%)	56	40-75
Lymphocytes (%)	31	20-35
Monocytes (%)	10.9	1.7-9.3
Eosinophils (%)	1.7	1-4
Liver function tests		
Albumin (g/L)	47	38-54
Bilirubin (total) (μmol/L)	24	5-21
Bilirubin (direct) (μmol/L)	10	0-5
Bilirubin (indirect) (μmol/L)	14	<18
Iron profile		
Iron (μmol)	9	9.0-30.4
Transferrin (g/L)	1.29	2.5-3.8
Transferrin saturation (%)	27	15-33
Ferritin (μg/L)	1237.1	7-282

**Figure 2:**
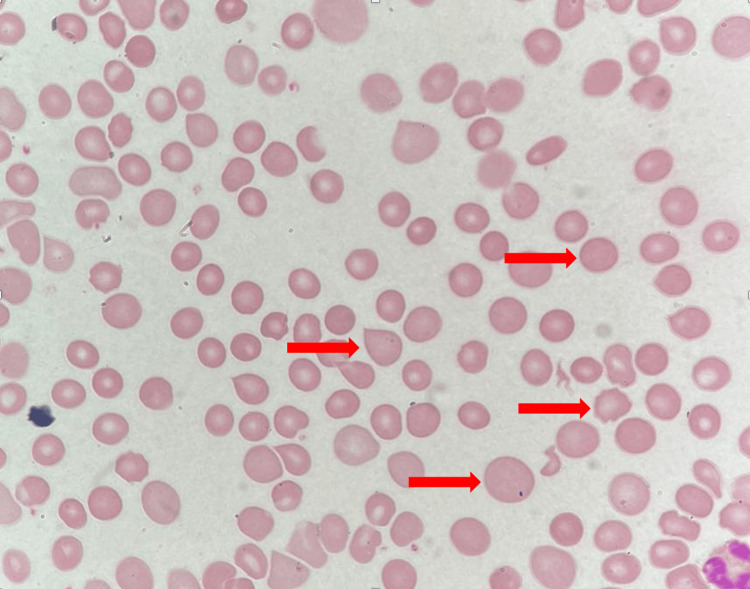
Peripheral blood smear Anisocytosis, the presence of macrocytes, tear drop cells, burr cells, polychromasia and spherocytes were noted (arrows).

**Figure 3:**
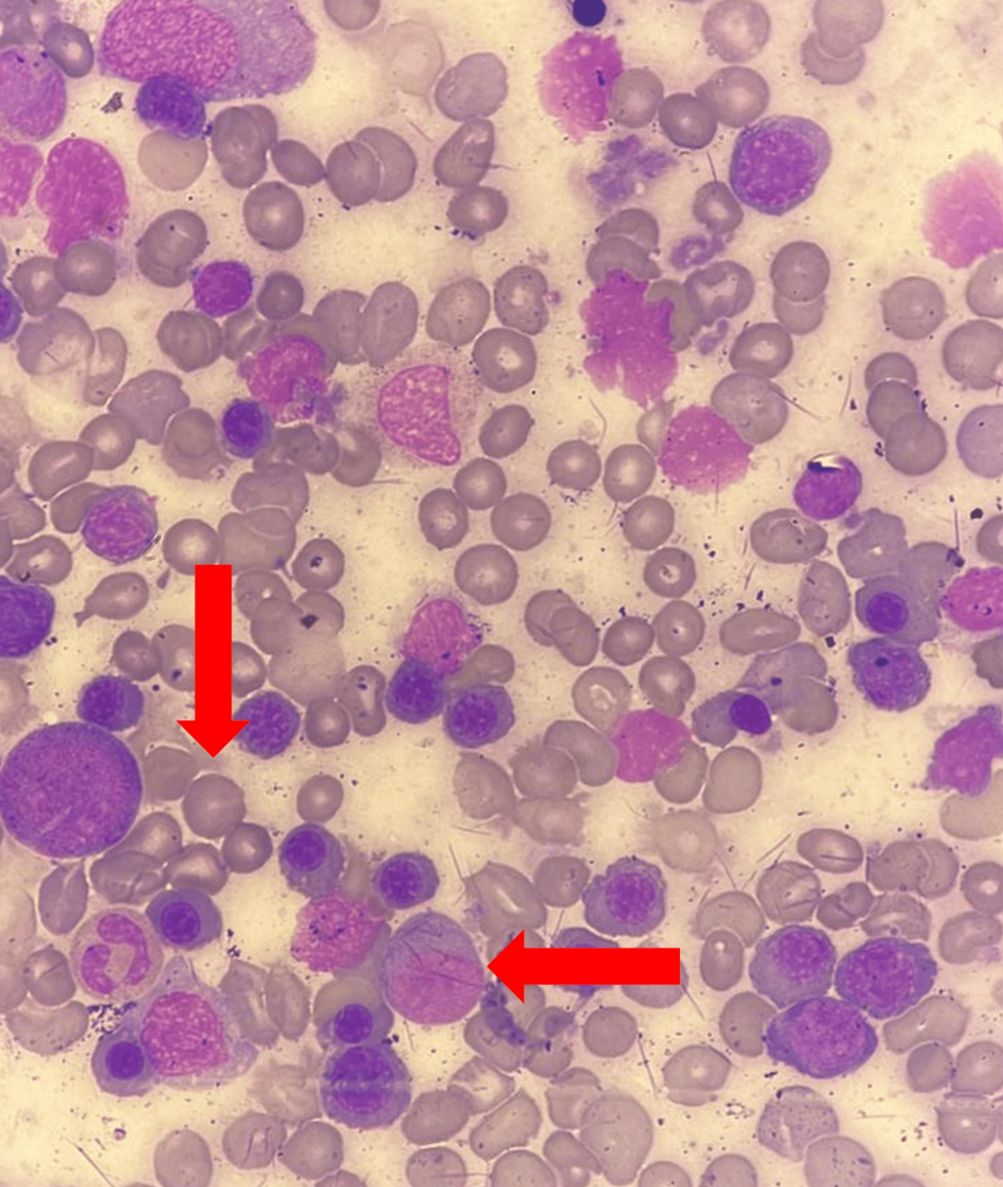
Bone marrow aspirate Hypercellular marrow with the presence of prominent erythroid hyperplasia accompanied by megaloblastic changes (arrows).

The peripheral blood smear demonstrated significant abnormalities, including anisocytosis, the presence of macrocytes, teardrop cells, burr cells, polychromasia and spherocytes (Figure 2). These findings are indicative of hemolytic anemia [3]. Bone marrow aspiration showed hypercellular marrow with increased cellularity and the presence of prominent erythroid hyperplasia accompanied by megaloblastic changes, suggesting an active compensatory response to ongoing red cell destruction (Figure 3). To further investigate the underlying cause, whole-exome sequencing was performed and identified a homozygous likely pathogenic variant in the GPI gene. This genetic finding confirms the diagnosis of autosomal recessive non-spherocytic hemolytic anemia caused by GPI deficiency.

**Figure 4:**
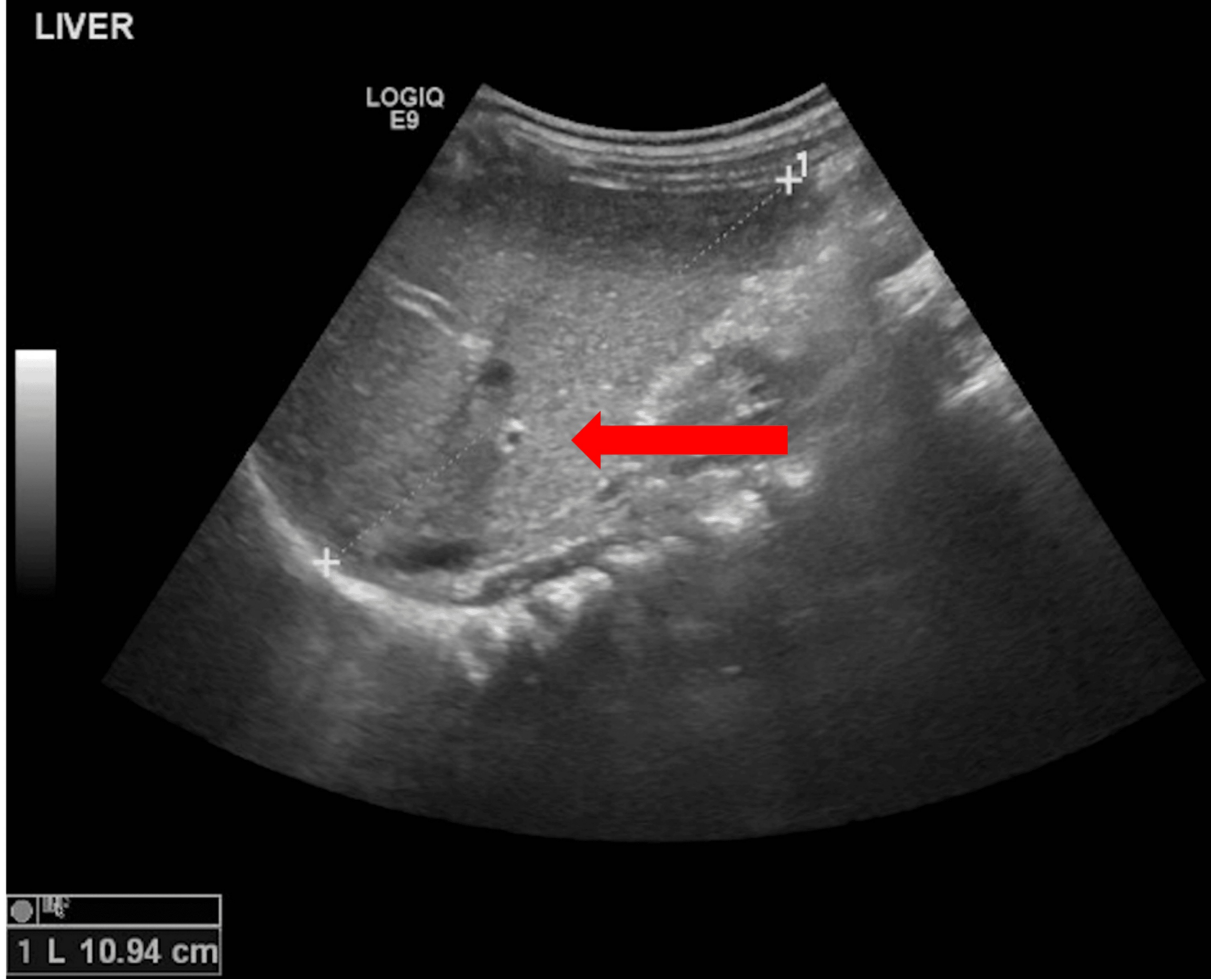
Ultrasound of the abdomen Ultrasound image showing hepatomegaly with the liver measuring around 11 cm along its long axis (arrow).

In addition to the laboratory investigations, radiological imaging was also performed. An ultrasound of the abdomen and pelvis revealed hepatomegaly, with the liver measuring 11 cm along its long axis in the midclavicular plane (Figure 4). No focal lesions were identified on the scan.

Since her diagnosis, the patient has been maintained on a regular treatment regimen that includes folic acid, vitamin D supplementation, and deferasirox. She has also received multiple blood transfusions, administered only when clinically necessary, typically during episodes of anemia that are worsened or triggered by infections.

## Discussion

The diagnosis of GPI deficiency in pediatric patients often necessitates consideration of rare metabolic disorders, particularly in cases of unexplained hemolysis. In our case, the diagnostic evaluation included a series of investigations, such as peripheral blood smear, bone marrow aspiration, and genetic testing.

Clinical presentation and diagnostic approach

The peripheral blood smear in cases of hemolytic anemia typically reveals anisocytosis and poikilocytosis, with occasional bite cells and schistocytes, findings that are characteristic of GPI deficiency and other glycolytic enzyme disorders [3]. In our patient, the smear demonstrated anisopoikilocytosis, polychromasia, hypochromia, a few macrocytes and microcytes, along with some teardrop and burr cells. Rare spherocytes and an insignificant number of schistocytes (0.2%) were also observed. Additionally, giant platelets were noted, and white blood cells showed occasional typical neutrophils, all consistent with a picture of hemolytic anemia.

The bone marrow aspiration revealed a particulate aspirate with hypercellular particles and trails. Megakaryocytes were slightly increased, and granulopoiesis was active, with a relatively reduced blast count of 2%. Erythroid hyperplasia was noted, along with megaloblastic changes, including karyorrhexis, cytoplasmic blebs, and binucleated forms. Hematogones and eosinophils were prominent. Perl’s stain demonstrated +2 to +3 iron stores, with no ring sideroblasts observed. Overall, the findings indicated a hypercellular bone marrow aspirate with active hematopoiesis, compensated erythropoiesis, and megaloblastic changes. The absence of significant immature cells helped rule out primary hematologic disorders, including leukemia or myelodysplastic syndromes [4].

Based on the clinical and laboratory findings, a genetic etiology was suspected, and whole-exome sequencing was performed through CENTOGENE Laboratory, Germany. The results revealed a homozygous likely pathogenic variant in the GPI gene, consistent with a genetic diagnosis of autosomal recessive non-spherocytic hemolytic anemia due to GPI deficiency. The identified variant, c.1157G>A p.(Arg386His), results in an amino acid substitution from arginine to histidine at position 386. According to HGMD Professional 2021.3, this variant has been previously described as disease-causing for GPI deficiency by Walker et al., 1993 [5], Lin et al., 2009 [6], and Jamwal et al., 2017 [7]. ClinVar also lists this variant as pathogenic (Variation ID: 13640) [8]. It is classified as likely pathogenic (Class 2) according to the recommendations of CENTOGENE and the American College of Medical Genetics and Genomics (ACMG) [9]. This mutation has been previously reported in the literature and aligns with the clinical and enzymatic features observed in GPI deficiency.

In addition to the primary investigations, several other diagnostic tests can assist in confirming the diagnosis or assessing the severity of the disease. Performing a direct antiglobulin test (DAT) can be utilized to exclude immune-mediated hemolytic anemia. In case of GPI deficiency, DAT is negative, confirming non-immune hemolytic anemia, and excluding other possible etiologies of immune-mediated hemolytic anemia [10]. Flow cytometry may be used to rule out autoimmune causes of hemolysis by detecting autoantibodies against red blood cells, especially in cases of suspected immune-mediated hemolysis [11]. Elevated levels of indirect bilirubin and reticulocytosis are commonly observed in hemolytic anemias, offering additional evidence of ongoing red blood cell destruction [3].

Management and treatment

Currently, there is no definitive cure for GPI deficiency, and management remains primarily symptomatic. The focus of treatment is on controlling hemolytic episodes, preventing complications, and improving the patient’s quality of life. In our patient’s case, management included regular blood transfusions, which were necessary to maintain hemoglobin levels and address episodes of severe anemia. Management of GPI deficiency mainly focuses on supportive treatment, including blood transfusions, iron chelation therapy, and might require splenectomy in some cases [12].

Due to the risk of iron overload from long-term transfusion therapy, iron chelation therapy was initiated. The patient is currently receiving deferasirox, a chelating agent that helps mitigate the toxic effects of excess iron, thereby reducing the risk of complications such as cardiomyopathy and liver dysfunction [13]. Although not yet indicated, splenectomy may be considered in the future if the patient develops significant splenomegaly or if transfusion requirements increase. Splenectomy has been shown to reduce hemolysis in GPI deficiency, thus reducing the transfusion requirement [1]. However, patients who have had a splenectomy carry the added risk of post-splenectomy infections [14].

Genetic counseling was provided to the family, as GPI deficiency follows an autosomal recessive inheritance pattern. This counseling addressed the implications for future pregnancies and emphasized the importance of carrier screening among family members [2]. Supportive care measures have also been implemented, including nutritional support and regular monitoring of iron levels, to prevent complications associated with chronic anemia and iron overload.

The prognosis for patients with GPI deficiency is generally favorable when the condition is managed appropriately. Although chronic hemolysis may persist, patients can often lead relatively normal lives with consistent blood transfusions, iron management, and close medical follow-up [12]. Lifelong monitoring is essential to detect and manage potential complications such as iron overload and splenic sequestration.

## Conclusions

This case highlights the importance of considering rare enzymatic disorders like GPI deficiency in pediatric patients with unexplained hemolytic anemia. Early diagnosis through enzyme activity assays and genetic testing is crucial for guiding appropriate management and preventing unnecessary investigations. As research into gene therapy and enzyme replacement advances, it offers hope for future therapeutic advancements.
